# Beyond barriers: a qualitative TDF and COM-B analysis of participation in active ageing programmes

**DOI:** 10.3389/fpubh.2026.1952870

**Published:** 2026-09-25

**Authors:** Nur Adilah Faizal, Benedict Wei Jun Pang, Yee Sien Ng, Yew Yoong Ding, James Alvin Yiew Hock Low, Lay Khoon Lau

**Affiliations:** 1 Geriatric Education and Research Institute, Singapore, Singapore; 2 Singapore General Hospital, Singapore, Singapore; 3 Tan Tock Seng Hospital, Singapore, Singapore; 4 Lee Kong Chian School of Medicine, Nanyang Technological University, Singapore, Singapore; 5 Khoo Teck Puat Hospital, Singapore, Singapore

**Keywords:** active ageing, behavioural determinants, capability, motivation, opportunity, self-efficacy

## Abstract

**Introduction:**

Global populations are ageing rapidly, underscoring the need for strategic policy responses such as active ageing programmes (AAPs). This study examined the barriers and facilitators influencing participation in the Live Well, Age Well AAPs among both participants and non-participants, to inform future programme refinements.

**Methods:**

Purposive sampling was employed to recruit older adults aged 50 years and above from diverse participation levels, non-participants, and dropouts from the programme. A total of 28 adults (23 females and 5 males; mean age: 72.8 ± 7.1 years) were recruited, and semi-structured interviews were conducted to gain insights from participants. The analysis was guided by the Theoretical Domains Framework (TDF) and Capability, Opportunity, Motivation – Behavior (COM-B).

**Results:**

Analysis of interviews with 28 participants revealed motivation as a central aspect of programme participation, with capability and opportunity identified and discussed in relation to motivational factors. Willingness to participate was driven by intentions to enhance knowledge, manage well-being, and develop digital skills. Health concerns about age-related ailments motivated engagement, though some reported physical discomfort deterred participation. Self-confidence was undermined by health conditions and negative perceptions about ageing. Technology-related fears, particularly regarding scams, combined with low digital proficiency heightened anxiety. Memory difficulties and limited English literacy further impeded engagement. Environmental factors including time availability and geographical accessibility were key determinants. Social influences operated alongside environmental factors, with family encouragement promoting engagement whilst discouragement created participation barriers.

**Discussion:**

This study demonstrates how TDF and COM-B models can guide theory-informed interventions for AAPs, revealing that participation involves multiple interrelated determinants requiring targeted approaches across capability, opportunity, and motivation domains.

## 1 Introduction

Population ageing, driven by declining fertility and mortality rates, has resulted in significant increase in the number of older adults globally. The World Health Organisation projects that one in six individuals globally will be aged 60 years and above by 2030 (1). This demographic transformation necessitates strategic policy response that enhance older adults’ quality of life, thereby reducing potential healthcare costs.

Active ageing programmes (AAPs) have been implemented globally in response to population ageing, though with considerable structural variation. A recent scoping review (submitted manuscript) conducted by the study team found that these programmes commonly address three core determinants – behavioural (physical activity, healthy eating), personal (cognition/psychological) and social determinants. Although a broad spectrum of programmes exists, participation among older adults fluctuates markedly, with perceived benefits and individual preferences acting as key determinants of engagement (2). While much attention has focused on older adults who actively engage in programmes, it is equally important to consider those who are aware of the offerings but remain reluctant to participate, as well as those who have dropped out of the programmes. Even in contexts where participation in certain programme component is mandatory, engagement often varies across modules, and overall adherence remains suboptimal (3, 4).

A particular area of concern is digital skill acquisition as older adults are commonly observed to display resistance or uncertainty when faced with learning new digital capabilities and processes (5). Poor digital literacy poses significant risks, as rudimentary digital skill could lead to social exclusion (6) with consequent adverse health outcomes (5). This participation gap is observed in Singapore, where while 93.4% of older adults seek digital literacy assistance from family members, only 44.6% engage in formal training workshops (7). However, with the demographic shifts in old age dependency ratio from 16.2 seniors aged 65 years onwards in 2015 to 26.5 in 2024 per 100 residents aged between 15 to 64 (8), the sustainability of seeking help from family members is questionable.

The low uptake of formal digital literacy programmes not only reflects barriers specific to digital skills but also highlights broader challenges older adults face in participating in AAPs. Language barriers have been identified as a significant barrier to older adults’ participation in some contexts. For example, some older adults were reported to have discontinued attendance at senior centres or programmes because language barriers made it difficult to fully engage with activities conducted in non-native languages (9). In Singapore, educational attainment presents a notable consideration, as despite improvement from 79.6% of older adults holding below-secondary qualifications in 2010 to 61.7% in 2020, a substantial segment remains affected (10). This demographic reality raises concerns on the potential of language-related obstacles in participation, including in digital literacy initiatives, which are mostly conducted in English and Chinese. The intersection of educational attainment and language proficiency may therefore represent a compounding barrier to engagement in AAPs among older adults.

Nevertheless, language barriers may not be universally applicable across all settings. For example, another Asian study examining social participation among older adults identified physical limitations, time constraints and chronic conditions were the key barriers to participation, with language playing a minimal role (6). This highlights that while language can be a significant obstacle in some contexts, other factors such as health, time, and mobility may equally shape participation outcomes.

Practical considerations, such as transportation and programme scheduling can also limit older adults’ participation. In some settings, particularly rural areas, distance to programme centres and limited public transportation significantly hinder access. Even in urban environments, unequal spatial distribution of programmes can create accessibility disparities (11). Singapore’s compact geography presents a different circumstance, with 223 active ageing centres (AACs) operating island-wide thus, facilitating convenient access for most older adults (12). However, convenience solely does not guarantee participation, spotlighting the need to examine other determinants that influence engagement rates beyond mere accessibility.

With the complex interplay of factors influencing AAPs participation which includes digital literacy challenges, language barriers, educational disparities and accessibility issues, understanding these dynamics within Singapore’s unique context is essential. While prior studies have identified individual barriers and facilitators, fewer have systematically interpreted these findings using behavioural science frameworks to inform intervention design.

Accordingly, this study aimed to examine the barriers and facilitators to participation in multi-component AAPs, specifically Live Well, Age Well (LWAW) among adults in Singapore. Importantly, this study extends existing literature by contextualising qualitative insights with theory-driven frameworks, specifically the Theoretical Domains Framework (TDF) (13) and the Capability, Opportunity, Motivation - Behaviour Model (COM-B) (14). As a voluntary and complimentary programme, LWAW includes programmes that span six components: Live Active, Live Assured, Live Enriched, Live Happy, Live Nourished and Live Prepared, with the aim of improving the physical, mental and social health of middle- to older-aged adults in the community (≥50 years old), as well as enhancing their health, digital and financial literacy, and overall quality of life. For this study, we evaluated four components – Live Active, Live Enriched, Live Happy and Live Prepared. LWAW provides a relevant context within Singapore to explore how demographic characteristics, multilingual considerations, and resources availability shape participation patterns in this unique urban setting. By mapping lived experiences onto behavioural constructs, this study offers a theory-informed understanding of participation that can support the development of more targeted and effective active ageing interventions. The research question was what barriers and facilitators influence participation in LWAW among adults in Singapore, and how these barriers and facilitators can be understood using the TDF and COM-B frameworks.

## 2 Methods

### 2.1 Study design

This study adopted a qualitative approach to explore participants’ perspectives on barriers and facilitators to participation in multi-component LWAW programme. Semi-structured interviews were conducted to allow participants to describe their experiences and perspectives. A deductive approach guided the analysis, with the TDF used as the theoretical framework for examining behavioural determinants of participation (13).

### 2.2 Participants

A purposive sampling approach was employed to recruit older adults (hereafter referred to as “participants”), aged 50 years and older, with varying levels of LWAW programme engagement from multiple community sites, including AACs and community clubs, where LWAW was implemented either as individual programme components or in combination. Potential participants were approached and invited to take part in the study by researchers during on-site running of the AAPs or through the support from community partners or programme implementers. Interested participants subsequently engaged with the researchers to better understand the nature of the study, and informed consent was obtained prior to their enrolment in the study. Three distinct participant groups were recruited based on engagement levels: participants (i.e., those who had engaged with one, two, three, or four components), non-participants, and dropouts from the AAPs. Non-participants were recruited from adults visiting the AACs who had access to the LWAW programme but did not participate. Barriers and facilitators were examined in relation to the individual LWAW component(s) in which participants had engaged, such that participants who had participated in the same component contributed to the understanding of barriers and facilitators specific to that component. Participants who had engaged with multiple components provided insights into their experiences across these components, as well as barriers to participation in other LWAW components. This approach allowed for a comprehensive understanding of diverse experiences and perspectives related to programme uptake, engagement and discontinuation. Given the heterogeneous participation profiles, saturation was considered across the range of participant experiences, with recruitment continuing until subsequent interviews yielded no substantively new barriers or facilitators relevant to participation in LWAW (15). This study has obtained ethics approval from National Healthcare Group Domain Specific Review Board (Reference number: 2024/00189).

### 2.3 Data collection

Semi-structured interviews were conducted to elicit responses from the participants, which were guided by an interview guide (refer to Supplementary file 1), with flexibility to refocus questions for more information from interviewers. The guide was developed primarily in accordance with the study objectives and informed by input from relevant stakeholders. The interviews explored participants’ exposure to the different LWAW components, and their current lifestyle practices, including exercise, healthy eating, and digital adoption. They also examined programme adoption and acceptability, including participants’ experiences, perceived barriers and facilitators, and perceived benefits, particularly in relation to lifestyle changes. Feasibility was explored through questions about challenges encountered and strategies used to overcome these challenges. Finally, participants were invited to suggest ways in which the programme could be improved. Open-ended questions and follow-up probes were used to allow participants to elaborate on their experiences and raise issues beyond those anticipated in the interview guide.

The interviews were conducted by four researchers—two PhD holders with additional formal training in qualitative methodology, one master’s degree holder in public policy and a bachelor degree holder in sociology. No prior relationship was established between the researchers and the participants. The average duration of the interview was 37 min, which was in line with the expected range of 30 min to an hour (16). The interviews were audio-recorded. We reported the study methods following the Standards for Reporting Qualitative Research (17) (refer to Supplementary file 2).

### 2.4 Data analysis

Audio recordings from the semi-structured interviews were transcribed by professional transcribers. For interviews conducted in Chinese, the recordings were translated into English during transcription. Transcribed files were verified for accuracy against the respective audio recordings. A directed content analysis was conducted using the TDF (version 2) (13) as the *a priori* coding framework in NVivo (version R1) (NVivo, n.d.). Two study members (the first and the last authors) were involved in the coding process. Both independently coded six transcripts before collaboratively reviewing and refining codes through discussion. Subsequently, the remaining transcripts were analysed independently, with 11 transcripts assigned to each coder. The definitions and descriptions of the TDF domains provided by Atkins et al. (13) guided the coding. Text relating to participation in the LWAW programme was included in the coding. Statements relating to other programmes other than LWAW were excluded. Relevant participant excerpts were classified to the closest-fitting TDF domain based on the meaning and context of the participants’ accounts. Where findings were not clearly represented in or explicitly covered by the coding guidance of Atkins et al. (13), a supplementary study-specific coding guideline was used to support consistent assignment to TDF domains. This guideline was iteratively developed and continuously updated throughout the coding process as new or ambiguous findings emerged.

Regular meetings were held to resolve any ambiguities during the analysis of these transcripts. In the final review, both members examined coding for all TDF codes and re-categorised any segments that had been initially coded differently. The two coders had different disciplinary backgrounds in physiotherapy and sociology, respectively, which provided complementary perspectives during the interpretation of participants’ accounts. Their differing perspectives, together with independent coding and iterative discussion, provided opportunities to critically examine interpretations and minimise the influence of individual assumptions on coding and categorisation.

To explore relationships between codes, a matrix coding query using the ‘Near’ function in NVivo (NVivo, n.d.) was conducted, allowing identification of instances where TDF domains appeared in close textual proximity. Directionality was interpretively inferred from the semantic content and context of participants’ accounts rather than from textual proximity or word order alone. This approach took into consideration the varying educational backgrounds of participants and the ways in which older adults may verbally express their experiences, which may not always follow conventional grammatical structures. This was particularly relevant for interviews conducted in Chinese, where differences in linguistic structure could affect the ordering of concepts in the English transcripts. Therefore, the apparent direction of relationships between TDF domains was interpreted based on the meaning conveyed in participants’ accounts and the contextual relationships between domains.

The social role and identity domain was excluded from this analysis as no participant excerpts were coded to this domain. A mapping of identified barriers and facilitators to the TDF domains is provided in Supplementary file 3. The remaining TDF domains were then mapped onto the COM-B model (18, 19) to facilitate interpretation and discussion of the findings. This two-step approach allows behavioural determinants to be first identified in details using TDF, and then organised within COM-B to provide a structured framework for understanding how capability, opportunity and motivation interact to influence participation. Framing the findings in this way highlights key influences on behaviour and their potential implications for intervention development.

## 3 Results

Table 1 shows the demographics of participants. A total of 28 participants, 23 females and five males with a mean age of 72.8 years old (SD = 7.1) were recruited: single component (*n* = 6), two components (*n* = 6), three components (*n* = 2), four components (*n* = 2), non-participants (*n* = 4) and dropouts (*n* = 8) (Table 2). The analysis of interviews with 28 participants revealed several key barriers and facilitators to their engagement in the LWAW programme. Motivation emerged as a prominent factor in participants’ accounts of participation. Capability- and opportunity-related factors were also identified and were frequently discussed in relation to motivational factors, suggesting interconnections among the COM-B components in shaping participants’ engagement in the programme (Figure 1). The relationships among the TDF domains and COM-B components described in the findings represent interpretive patterns identified from participants’ accounts, reflecting how these domains were discussed and interconnected within the qualitative data.

**Table 1:** Demographics of participants.

Characteristics	Participants [*n*(%)]
Age group (years)	
50–59	2 (7.1)
60–69	7 (25)
70–79	15 (53.6)
80 and above	4 (14.3)
Gender	
Male	5 (17.9)
Female	23 (82.1)
Ethnicity	
Chinese	28 (100)
Education	
No formal education	2 (7.1)
Primary	10 (35.7)
Secondary	9 (32.1)
Up to diploma	5 (17.9)
University	2 (7.1)
Housing	
Public housing (1–2 rooms)	6 (21.4)
Public housing (3 rooms)	3 (10.7)
Public housing (4–5 rooms)	17 (60.7)
Private properties	2 (7.1)
Living arrangement	
Alone	6 (21.4)
With spouse	7 (25)
With spouse and children	6 (21.4)
With children	7 (25)
Others	2 (7.1)

**Table 2:** Levels of programme participation.

Programme participation	Participants [*n*(%)]
Dropouts	
Live Active	8 (28.6)
Single component	
Live Active, Live Enriched, Live Happy or Live Prepared	6 (21.4)
Two components	
Live Active + Live Enriched	6 (21.4)
Live Active + Live Happy
Live Enriched + Live Happy
Live Active + Live Prepared
Three components	
Live Active + Live Enriched + Live Happy	2 (7.1)
Live Active + Live Enriched + Live Prepared
Four components	
Live Active + Live Enriched + Live Happy + Live Prepared	2 (7.1)
Non-participants	4 (14.3)

**Figure 1:**
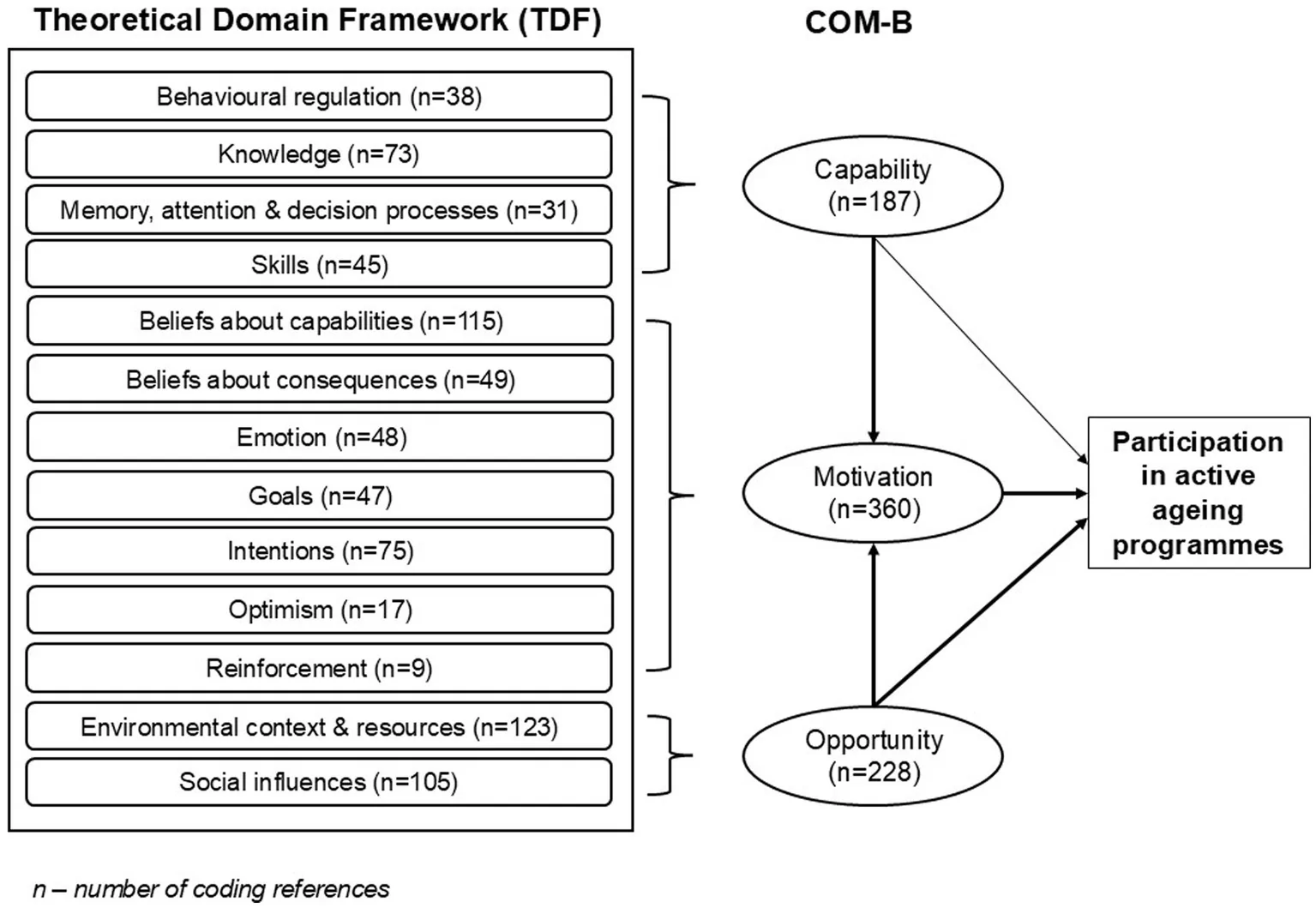
Mapping of TDF domains to the COM-B model.

### 3.1 Motivation

#### 3.1.1 Intention and goals

More than half of the participants expressed a willingness to participate in the programmes, driven by specific intentions. These included enhancing their knowledge through educational talks, managing well-being through exercise programmes and developing mobile phone proficiency through digital workshops. Some participants already possessed basic knowledge but were motivated to attend programmes to gain additional information or to reinforce existing understanding, particularly in relation to health and well-being. As one participant shared.

*“Existing knowledge…er…er…I would say may be to me is enough, but actually there are more than that, which I might not know, you see. So when given this type of talk, we can gain more.”(P14, Male, 74 years old).*

In addition, participants expressed a desire to keep up with rapid technological advancements, which encouraged their participation in digital workshops. Overall, the intention to improve knowledge and well-being through these targeted activities was central to their decision to join, highlighting intention as a key facilitator of engagement.

Building on these intentions, two-thirds of the participants identified knowledge acquisition as a key goal of their programme participation. Educational talks were valued as sources of new or updated information while exercise programmes were pursued to improve physical fitness and learning skills to enhance their strength, balance and coordination. These clearly defined goals were important to participants’ decisions to engage in the programmes.

*“I like to attend all this talk, to get more knowledge ah. Ya, it’s nothing wrong if you got more knowledge when you…all of a sudden you…you met some dementia person, you know how to take care.” (P21, Female, 76 years old).*

#### 3.1.2 Beliefs about consequences

Half of the participants cited age-related ailments that could progressively deteriorate without intervention as key motivators for programme participation. They described muscle deterioration, physical discomfort from inactivity, poor blood circulation due to clogged vessels and lethargy as consequences of not engaging in exercise. These anticipated negative outcomes motivated them to participate in the programme as a means of mitigating adverse health effects and preventing further health decline. However, a small number of participants reported that engaging in physical activity exacerbated their bodily pain or physical discomfort, which in turn reduced their willingness to participate in the programme.

*“If I continue to join ah, I bring misery to myself mah” (P27, Female, 74 years old).*

*“I tend to have pain here and there. Ah so I – a lot of thing I dare not participate is due to my health problem.” (P10, Female, 63 years old).*

A few participants highlighted the benefits of improving their technological skills as factors that encouraged participation in the digital programme. They described several perceived advantages supporting their engagement, including avoiding long bank queues through online banking, receiving additional benefits for online transactions, maintaining digital competency to reduce feeling of exclusion, and increasing awareness to safeguard against fraudulent activities.

#### 3.1.3 Beliefs about capabilities

Several participants reported that existing health conditions reduced their self-confidence, in turn their motivation to participate in the programmes. Some of the health conditions mentioned were visual impairment and weakness of the upper and lower extremities. Additionally, two participants expressed caution in avoiding over-exertion following surgical procedures. Such health-related concerns appeared to undermine their self-efficacy, thereby lowering their likelihood of participation.

*“Basically it’s my pain, ya. Then because my- my stretching ah, maybe I do wrongly or what lah. The-the joint kind of pain ah, that made me er…really wanted to do it, but then…(laughter). ‘The heart is willing but the flesh is weak’ (translated from Chinese). (P10, Female, 63 years old).*

Beyond these direct health-related impacts, beliefs about capabilities were also intertwined with other psychological domains of the TDF, such as optimism and emotion, creating complex relationships that further influenced participants’ programme participation decisions, as discussed in the following paragraphs (Figure 2).

**Figure 2:**
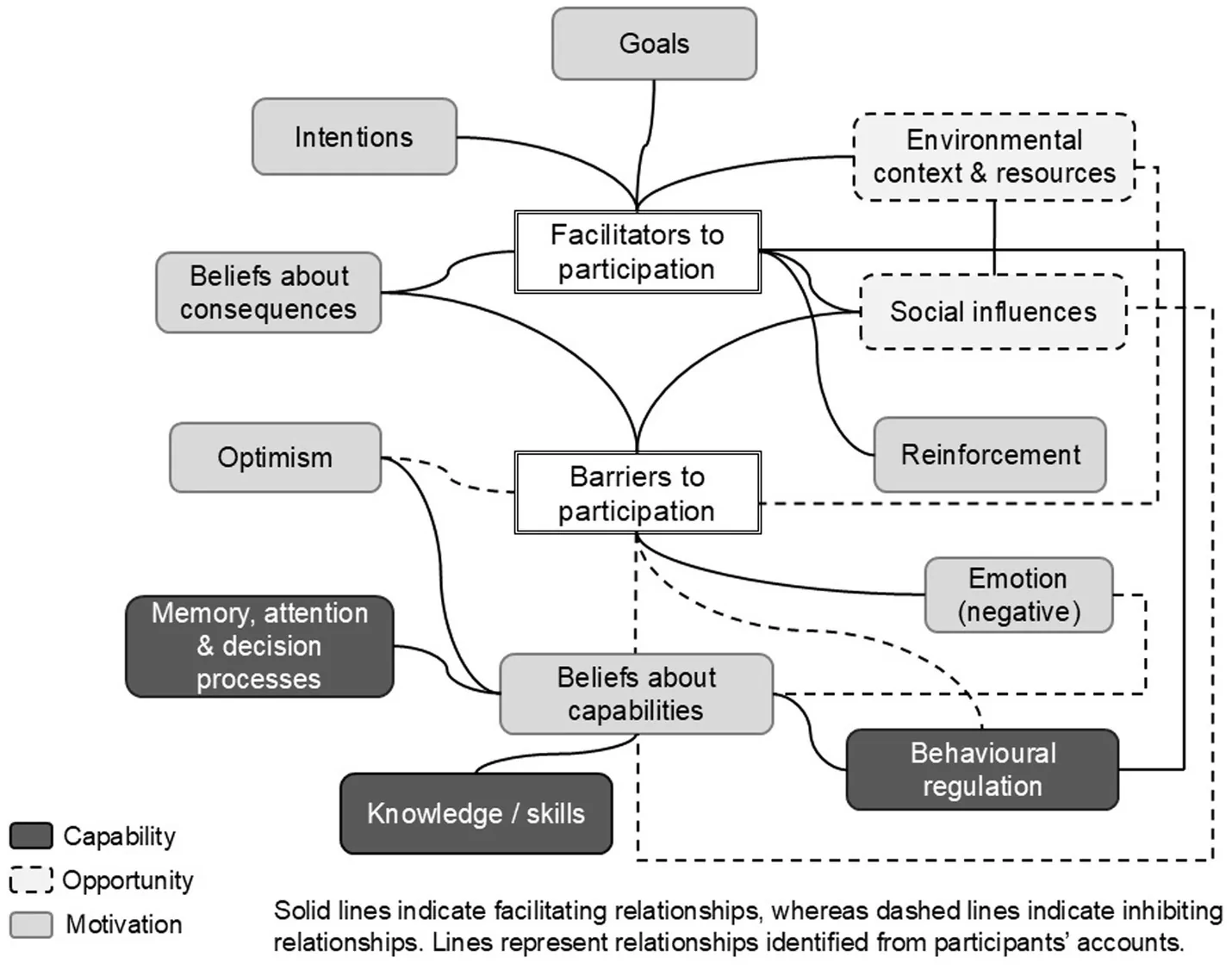
Interactions between TDF domains.

#### 3.1.4 Pessimism and weakened beliefs about capabilities

Building on the influence of health-related limitations on self-efficacy, some participants expressed negative age-related self-perceptions that reflected internalised self-ageist attitudes, which hindered their willingness to participate in the programmes. They expressed pessimistic views about ageing and their own learning capacity, or perceived no need to learn, describing difficulties in acquiring new knowledge, and in some cases, perceiving learning as burdensome or unnecessary at their stage of life. These age-related beliefs were expressed alongside doubts about their capabilities, and reduced willingness to participate in the programme.

*“Because I’m also old already, still want– still want them to increase what? You look at my body, I also can’t do and keep up mah!” (P27, Female, 74 years old)*

#### 3.1.5 Weakened beliefs about capabilities and heightened negative emotions

A few participants reported concerns related to technology use, particularly fear of fraudulent schemes, coupled with limited confidence in their technological abilities. In addition, few participants expressed apprehension about using digital devices due to visual impairments and reported reduced confidence, and greater reliance on others. These accounts reflected heightened caution when using mobile phones, with participants describing concerns about scams, limited technological proficiency, and perceived vulnerability.

*“It’s not that I don’t want to learn. No confidence. Now a lot of people ask me to go and learn. It’s not that I don’t want. Because my hand is sometimes very fast. So CDC I don’t want to put in my handphone. I want the voucher. My reason is because sometimes I can’t see properly. Two, sometimes I’m also very fast. I press quickly $2 I put $20.” (P4, Female, 70 years old).*

Some participants also described difficulties in learning new skills, with the pressure to keep up leading to feelings of stress and discouragement when they were unable to grasp concepts. These experiences contributed to negative emotional responses, which alongside reduced self-efficacy, further deterred their participation in the programme

“Because sometimes when you’re unable to learn it hor, you have this feeling, aiyo, die! What the hell is this? Why am I so stupid, I don’t know? Aiya, don’t go and think anymore lah! Don’t know then don’t know lah!” *(P11, Female, 76 years old)*

Overall, the findings suggest an interplay among beliefs about capabilities, pessimism, and negative emotions in participants’ accounts of programme participation. Participants described health-related limitations and age-related stereotypes in connection with doubts about their capabilities and pessimistic views about learning and personal competency. These perceptions of limited capability co-occurred with anxiety and fear, particularly in relation to technology use and potential scams. Collectively, these psychological factors contributed to barriers to programme participation.

### 3.2 Capability

#### 3.2.1 Memory, attention and knowledge barriers and beliefs about capabilities

The participants reported making input errors when using digital devices, which they attributed to visual impairments and anxiety-induced haste. These negative experiences were accompanied by resistance towards digital technologies, including internet banking, and reduced motivation to use mobile phones or engage in technology-related programmes. Additionally, participants described difficulties with retaining information and expressed reluctance to learn.

*“Because I don’t want to- because I would forget. Ah, because you- you- people explain and explain and you forget yet again. Right or not? I don’t want to learn lah.” (P19, Female, 76 years old)*

To cope with these challenges, few participants attempted strategies to reinforce learning, such as taking screenshots of workshop contents or borrowing hardcopies of post-workshop notes for later reference.

*“Ah but memory counts. After a while I forget. So I’ll – when I attend, I always take screenshot.” (P8, Female, 66 years old)*

Several participants had limited English literacy proficiency and low levels of education, which affected their confidence in processing the information delivered in the programmes. They reported difficulties in understanding sessions conducted in English, with low educational levels posting additional challenges to comprehension. One participant highlighted that although some workshops were conducted in Chinese, most web interfaces are in English, requiring participants to understand the language and thus presenting a persistent barrier for those with limited English proficiency. These dual barriers impeded knowledge acquisition and, in turn deterred participation.

*“They use English to talk and they also explain in Chinese. But when it comes to operating the phone, you still need to know how to read English. So I…I cannot cope lah. Because I only studied until primary school mah. I completely can’t read English one.” (P3, Female, 76 years old)*

#### 3.2.2 Belief about capability and behavioural regulation

Among participants in the exercise programmes, only a small proportion consistently performed the recommended home-based exercises or incorporated what they had learned into their existing routines. Most participants were less inclined or consistent in doing so, with some expressing concerns about whether they were performing the exercises correctly and indicating a preference for guidance from trained personnel. Factors such as self-discipline, family commitments, and a preference for group-based exercise settings were also identified as influencing adherence. The majority of participants reported that they had already been following a healthy diet prior to joining the health literacy workshop. However, one participant reported paying closer attention to nutritional needs when planning meals after attending.

*“So I find- from there ah, because they got- they- they- they got er…do…from warm-up, and then er do the- the whole proper share. Proper kind of programme ah. So I find it’s more appropriate for- for people like me ah. You know? Like I can really follow properly”*

*“Ah, where else… I do at home ah, I can anyhow do more. Then I follow the YouTube I can do wrongly also. (Laughter) That’s why you know, I find if- if- with coaching ah, coaching.” (P10, Female, 63 years old)*

### 3.3 Opportunity

#### 3.3.1 Environmental context and social influences

The majority of participants identified time availability and geographical accessibility as key factors influencing programme participation. Participants were more likely to engage when sessions were scheduled conveniently and held at easily accessible locations, particularly within their residential complexes at AACs. Conversely, several participants reported barriers such as caregiving responsibilities and work schedule conflicts that limited their ability to participate. Thus, temporal availability and location convenience emerged as dual determinants of programme participation.

*“I just need to have the time, given my schedule. Because my time is usually taken up by all these appointments, that I have no choice.” (P15, Male, 84 years old)*

These environmental factors operated alongside social influences, which either encouraged or discouraged participation in the programme. Accessible timing and convenient locations, combined with encouragement from AACs staff and friends, facilitated engagement in the AAPs. Some participants viewed this as an opportunity to expand their social networks. By contrast, discouragement from friends and family, particularly family concerns about fall risks when travelling to distant venues deterred participation, illustrating how social influence can outweigh individual motivation when combined with geographical barriers.

*“Because all of them are working. If they- if I- if I want it, they have to take off, you see.”*

*“Er, public transport for me is ok. But I have er relatives that taking public transport ah, fall on the…bus ah. Then paralyse. So they say that they scared. My children are afraid. They say don’t take public transport. They will always bring me. Unless- or else I take Grab, you know.” (P1, Female, 85 years old)*

#### 3.3.2 Social influence and beliefs about capabilities

A few participants reported that family members discouraged them from using digital devices, emphasising caution due to unfamiliarity. This approach fostered fear of making mistakes and undermined their confidence in using digital skills.

*“Later I press wrong…My daughter tells me “You don’t anyhow press ah. Don’t anyhow press.’ A lot of harassment also lor. I don’t dare. Because I don’t recognise English! So I don’t want (Laughter).” (P16, Female, 71 years old)*

Some participants also described family members assisting them with tasks instead, which was associated with greater reliance on family members and a reduced perceived need to acquire new skills independently.

*“If you have someone to rely on, of course you’ll rely on them first lah. Otherwise they will feel they have no job to do. (Laughter) They– they will feel that you don’t need them anymore. (Laughter) Right or not?” (P18, Female, 69 years old)*

Conversely, one participant shared the need to acquire new skills by attending the workshop, as the family member was perceived as less supportive and unwilling to help or teach them. Overall, the interplay between environmental accessibility and social support networks shaped participation decisions.

*“You want to learn from your children, a bit difficult. Because sometimes they teach you, they say ‘Like that like that like that’. Then you don’t know they say, ‘So stupid!’ (Laughter) You know lah, like…let’s say maybe your father teach you. Your father ask you this then you say ‘Like that like that like that’. Then your father still cannot. You say ‘Wah! The old man very stupid!”*

*“I mean how long you’re going to live you don’t know mah. You use– don’t want to be backward lah,” (P5, Male, 75 years old)*

## 4 Discussion

This study aimed to examine the barriers and facilitators to participation in multi-component AAPs among adults in Singapore. It extends existing literature by using the TDF and the COM-B model to contextualise qualitative insights and demonstrate how multiple behavioural determinants interact to shape participation in AAPs. Previous research has applied TDF and the COM-B to identify barriers and facilitators to health-related behaviours in older adults, such as reducing sedentary behaviour (20) or engaging in physical activity programmes (19). These studies have highlighted the importance of domains such as knowledge, environmental context, social influences and motivation. Importantly, our findings further demonstrate that individual TDF domains may function as either facilitators or barriers depending on participants’ experiences and circumstances.

Building on this evidence, our study extends current understanding by illustrating the dynamic interplay between capability (e.g., health status, literacy), opportunity (e.g., accessibility, family support) and motivation (e.g., beliefs about capabilities, optimism/pessimism, emotional responses) in shaping participation in AAPs. Our qualitative findings further suggest that capability-related experiences were closely related to motivational factors that influenced programme participation.

Although previous studies have reported that capability directly influences participation in physical activity (21), our findings suggest that, within the context of AAPs, capability-related factors may be particularly relevant to motivation. Unlike exercise-only interventions, participation in AAPs involves a broader range of behaviours, including programme selection, learning and social engagement. Participants are required to identify appropriate programmes, understand educational content, communicate with facilitators and peers, and navigate registration processes. Capability limitations, including language proficiency, digital literacy, educational background and memory issues, appeared to shape participants’ beliefs about their ability to participate (self-efficacy) and the perceived relevance of the programme, as well as their motivation and willingness to engage.

Consistent with this interpretation, participants with limited English proficiency and lower educational attainment experienced challenges in comprehending programme content. Language barriers, particularly in programmes delivered solely in English, have been consistently reported as a barrier to engagement among older adults, especially in non-English-speaking contexts (22). In Singapore, the transition towards English-medium education occurred progressively from 1960s. Older adults who were educated before or during this transition may have had less exposure to English during their formal education (23). Compounding this challenge, most digital interfaces, including mobile applications and websites, are predominantly in English (24), making navigation difficult even when workshops are conducted in Chinese and the programme content is understood. Together, these findings highlight the importance of capability-related factors, including language, educational background and digital literacy, in participants’ confidence and perceived ability to engage in AAPs.

Motivation, particularly reflective motivation, emerged as a central aspect of engagement in the LWAW programme. Reflective motivation refers to the conscious process involved in forming intentions, setting goals and evaluating the value of actions (14). Most participants held clear aims such as enhancing physical wellbeing or acquiring and reinforcing knowledge, which align with literature showing that goal-directedness (25) and perceived benefits (26) are important for sustained behaviour in older adults. The largely group-based nature of the LWAW programme further supported participation, as participants expressed a desire to expand their social networks, reflecting the importance they place on social connectedness with friends (27). Participants were motivated by multiple factors, including improving physical health, pursuing lifelong learning, strengthening social connectedness and enhancing psychological well-being. Collectively, these motivations may have supported continued engagement, particularly when participants perceived the programme content as valuable and relevant to their personal needs. Hence, given the diverse needs of older adults, allowing them to choose the programme that best suits their needs and preferences may improve participation in AAPs.

Additionally, participants in our study demonstrated an awareness that non-participation in exercise could lead to progressive physical decline, including muscle atrophy, loss of muscular strength, reduced mobility and an increased risk of falls and their associated consequences. These concerns were reflected in participants’ motivation to engage, as maintaining health and functional ability was a key priority (26, 28). Participants also valued the broader benefits of exercise, reporting improvements in energy levels, reduced fatigue and enhanced psychological well-being. Together, these findings suggest that adults’ motivation to engage in AAPs is driven not only by the avoidance of negative outcomes but also by the pursuit of positive physical and psychological health benefits. While exercises have been shown to reduce pain intensity through psychological mechanisms (29), a few participants experienced discomfort or pain during exercise, and described risk-averse behaviour that limited their participation, consistent with previous findings (30).

Despite recognising the benefits of AAPs, some participants remained reluctant to participate due to negative self-perceptions that associated ageing with a reduced ability to learn. In our study, these perceptions were reflected in lower levels of self-efficacy, with some participants expressing concerns about memory decline and doubting their ability to remember new information. This is consistent with existing literature linking lower self-efficacy to reduced participation (31, 32). A few participants also expressed apprehension towards formal classroom-based learning environments. This finding may help explain why participation in lifelong learning initiatives remains relatively low among older adults despite the availability of supportive policies. For instance, in Singapore, SkillsFuture, a national initiative that provides credits to support lifelong learning and skills development, has seen relatively low utilisation among older adults, with only 32% of individuals aged 60 years and above using their credits, representing the smallest proportion among those aged 40 and above (33). Therefore, increasing awareness of programme benefits or providing physical infrastructure alone may be insufficient to promote engagement if age-related beliefs and perceptions about learning remain unaddressed.

Beyond beliefs about learning ability, some participants also expressed negative age-related self-perceptions that were consistent with internalised self-ageist attitudes, particularly in relation to their perceived ability or need to learn at an older age (34, 35). These beliefs were accompanied by doubts about their capabilities and reduced willingness to participate, suggesting an interplay between self-perceptions of ageing and one’s sense of capabilities. These findings can be considered alongside prior research describing passive perspectives on ageing, characterised by the acceptance of minimal daily functioning as sufficient rather than the pursuit of active engagement (28). However, contrasting evidence suggests that some older adults view ageing positively, perceiving it as non-limiting and remaining motivated to actively manage their well-being (36, 37). Collectively, this body of research underscores the critical role of self-perception of ageing in shaping motivation and, ultimately programme participation.

Reflective motivation also appeared to interact with automatic motivation, which encompasses emotion-driven processes such as fear, anxiety and stress that influence behaviour (14). Participants with lower self-efficacy often described heightened anxiety when faced with learning new skills or navigating technology challenges, suggesting an association between lower confidence and greater negative emotional responses. This aligns with existing evidence demonstrating that lower levels of self-efficacy, predispose individuals to greater anxiety and discouragement, perpetuating barriers to behavioural engagement (38) in the broader context of participation in AAPs.

Beyond participants’ perceptions of their capabilities, several external factors within the opportunity domain hindered programme participation. Opportunity is thought to affect behaviour directly and indirectly through motivation, highlighting how contextual factors can shape engagement (14). In our study, social opportunity was influenced by multiple factors. Participation in AAPs was often encouraged by staff at AACs and, in some cases, by friends, highlighting the importance of supportive social networks. At the same time, family dynamics could constrain engagement where reliance on younger family members for assistance reduced the perceived need to acquire skills, potentially reinforcing dependency patterns and limiting opportunity for digital literacy enhancement (39). We also found instances where well-intentioned, yet overprotective family members restricted participants from taking part in programmes, echoing findings that family concerns, such as fears about loss of consciousness during activity can generate anxiety and deter programme engagement (26). Together with previous research highlighting the important role of family support in older adults’ adoption of digital technologies (40), our findings extend this understanding by demonstrating that family influences are not uniformly supportive or restrictive but may facilitate or hinder participation depending on the type of assistance provided. Hence, efforts to improve older adults’ participation in AAPs should extend beyond older adults themselves. Engaging caregivers and family members may be important for addressing misconceptions, alleviating concerns, and fostering supportive environments that encourage participation while promoting greater independence.

Physical opportunity was constrained by time limitations due to family obligations such as caregiving responsibilities that hindered participants from attending programmes. Caregivers often faced difficulties balancing caregiving duties with programme participation (41). Additionally, the increase in the retirement age also contributed to physical opportunity barriers, as older adults remained in the workforce longer, resulting in reduced time availability for programme participation. Prolonged employment beyond the age of 65 can reduce participation among older adults (42). These findings highlight how broader societal and family responsibilities may constrain opportunities for programme participation, even among individuals who are motivated to engage.

This study advances the field by demonstrating how the TDF and COM-B model can be integrated to provide a theory-informed lens on participation behaviour in multi-component AAPs. Our findings highlighted that participation was shaped by multiple interrelated determinants, including capability, opportunity and motivation, rather than single barriers alone (43). The relationships among COM-B components suggest that interventions addressing one component may have implications for others. For example, enhancing capability through skill-building, digital literacy support or language-adapted materials may strengthen confidence and self-efficacy, which are aspects of reflective motivation while also reducing reliance on family assistance and supporting social opportunity. Similarly, addressing opportunity barriers through flexible scheduling, accessible digital platforms and supportive social networks may reduce environmental and social constraints that influence motivation. These findings may also provide considerations to inform policy development for AAPs by highlighting key behavioural determinants associated with participation.

Although the TDF and COM-B provide useful frameworks for organising and interpreting behavioural determinants, our findings also highlight that participation in AAPs is situated within broader relational and contextual circumstances. This broader interpretation aligns with the WHO framework for healthy ageing, which conceptualises functional ability as the interaction between an individual’s intrinsic capacity and the environments in which they live, highlighting the importance of supportive physical and social environments in enabling individuals to do what they value (44). While the TDF and COM-B were used to structure the analysis in this study, a social-ecological perspective provides a broader lens through which these findings can be contextualised across individual, interpersonal, organisational, community and societal levels (45). Viewed from this perspective, participation in AAPs should not be understood solely as an individual choice or motivation, but as behaviour embedded within the social and environmental contexts in which older adults live. This broader framing complements the TDF and COM-B by drawing attention to how relational and contextual conditions may enable or constrain the translation of individual capability and motivation into participation.

One potential intervention to address the observed relationship between beliefs about capability and negative emotion is the use of mastery experiences to strengthen participants’ self-efficacy. Mastery experiences involve engaging individuals in incremental, achievable tasks that allow them to experience success and build confidence in their capabilities (46). By structuring engagement in the AAPs around small, scaffolded steps and providing immediate positive feedback, participants may be more likely to perceive their progress and feel capable of handling new skills or technological challenges. This approach could address both aspects of the observed relationship: enhancing reflective motivation by reinforcing beliefs in personal competence and reducing automatic negative emotions such as anxiety and stress by making tasks predictable and manageable. Over time, repeated mastery experiences can disrupt the cycle of low confidence and heightened negative affect, promoting sustained behavioural engagement.

The insights from our study could be further mapped to the Behaviour Change Wheel to inform the selection of targeted intervention functions based on the behavioural determinants identified across the COM-B domains. For example, capability-related determinants may be addressed via training, education and enablement; motivation-related determinants through persuasion, modelling and incentivisation; and opportunity-related determinants via environmental restructuring and enablement (14). Overall, this study adds depth to the application of behaviour change theory in gerontological research by illustrating how TDF and COM-B can guide the design of theory-informed, multidimensional interventions to enhance engagement in AAPs.

Future research should focus on developing and evaluating evidence-based programme modifications that align with participants’ capabilities. Given that adults with lower self-efficacy or educational attainment may perceive programmes as overly challenging, tiered programme structures with graduated difficulty levels could enable engagement at an appropriate level. Programme adaptations tailored to adults with lower formal education can significantly enhance participation rates (47) as motivation is more likely to be translated into action when individuals possess the capability to engage (48).

Beyond individual capabilities, attention should also be given to the evolving demographic profile of older adult populations. As successive cohorts demonstrate higher educational attainment than current participants, research should examine how programme curricula and content can be systematically re-evaluated and adapted to match participants’ enhanced knowledge bases and evolving learning expectations. This includes investigating how programme planners can effectively monitor emerging trends and topics of relevance to future adult cohorts. Additionally, research should evaluate the impact of flexible delivery methods on participation accessibility. Studies should examine whether offering programmes across multiple timeframes and platforms including both physical and virtual formats improves engagement among caregivers and employed older adults who express interest but face logistical constraints.

Methodologically, future research could also benefit from sequential exploratory mixed-methods designs, combining qualitative depth with quantitative breadth (49). Insights from semi-structured interviews can guide the development of structured quantitative surveys to measure prevalence and distribution of participation barriers across larger, more diverse populations. Subsequently, the qualitative and quantitative findings could inform system dynamics simulation models developed through Group Model Building (50) with key stakeholders to explore interactions between barriers and identify leverage points for intervention. This integrated approach would provide a comprehensive understanding of participation patterns and support evidence-based strategies to enhance programme engagement across diverse demographic groups.

The present study has several strengths. The methodology enabled the collection of rich qualitative data, providing insights into the diverse experiences of participating and non-participating adults and allowing for a comprehensive understanding of both the facilitators that promote their participation and barriers that impeded them. Our study also recruited a diverse age sample, encompassing adults that were both retirees and actively employed as well as those with varying educational background ranging from no formal education to university-level attainment, thereby enhancing the breadth and relevance of findings. Importantly, participants contributed suggestions for programme improvements, offering practical directions to refine existing LWAW initiatives.

Several limitations should be noted. Firstly, despite recruiting participants of all genders, the sample was predominately female, reflecting the higher female participation rates typically seen in such programmes. Secondly, all participants were of Chinese ethnicity, limiting insights into the experiences of minority communities. Cultural differences in health beliefs, family dynamics, communication preferences and attitudes towards ageing may influence participation patterns differently across ethnic groups. Additionally, language barriers, cultural stigma around certain health topics or varying levels of trust in formal programmes may present unique challenges for participants from different cultural backgrounds that were not captured in this study. Lastly, participants’ recollections of their experiences and motivations may have been influenced by time elapsed since programme participation.

## 5 Conclusion

In conclusion, older adults’ participation in AAPs is shaped by a dynamic interplay of barriers and facilitators that influence engagement. Grounded in participants’ lived experiences, this study underscores the value of a participant-centred approach in refining existing programmes and designing future initiatives. By aligning programmes with older adults’ preferences, capabilities, and contextual realities through theory-informed adaptations, interventions can enhance both relevance and meaningfulness of engagement, strengthening the effectiveness of active ageing initiatives.

## Data Availability

The original contributions presented in the study are included in the article/Supplementary material, further inquiries can be directed to the corresponding author.
