# Supplementary material for: Beyond barriers: a qualitative TDF and COM-B analysis of participation in active ageing programmes
Source: Front Public Health. 2026 Sep 25;14:1952870. doi: 10.3389/fpubh.2026.1952870 (PMC13649756; doi:10.3389/fpubh.2026.1952870)
Supplement: Supplementary file 1 [file Table_1.DOCX]

Supplementary Material

# 1 Supplementary Data

## Supplementary 1. Interview guide

**Introduction**

1) Explanation of ethics, consent and confidentiality of interview and analysis.
2) Explanation of purpose of study.
3) Explanation of structure and duration of interview.

**Rapport building**

1) Involvement of the participant in the active ageing programme / any exercise programme on their own.
2) Current exercise and health behaviour, social network and social activities, IT or technology adoption in daily life (whichever applicable).
3) The exercise and health behaviours, social network and social activities, IT or technology adoption in daily life (whichever applicable), prior to the enrolment into the active ageing programme.

**Acceptability**

1) Participants’ experience about the active ageing programme.
2) Barriers and facilitators to uptake and/or adherence to the programme by participants.
3) The satisfactory level of participants about the programme.
4) The perceived benefits yielded from the participation in the programme.

**Feasibility**

1) Challenges faced in the active ageing programme.
2) The difficulty level perceived by the participant in carrying out the programme.
3) Strategies in overcoming the challenges faced and their respective reason(s).

**Sustainability and suggestion**

1) The plan to continue with their exercise or lifestyle after the programme?
2) Suggestion on ways to improve the active ageing programme.
3) Ways to engage older adults to take part in the active ageing programme.

**Others**

1) Other issue(s) related to participation in the programme.

**Ending**

1) Thank the participant for his or her time and information.

## Supplementary 2. Standards for Reporting Qualitative Research (SRQR)

|  | **Standards for Reporting Qualitative Research (SRQR)*** |  |
| --- | --- | --- |
|  | <http://www.equator-network.org/reporting-guidelines/srqr/> |  |
|  |  | **Page/line no(s).** |
| **Title and abstract** | |  |
|  | **Title** - Concise description of the nature and topic of the study Identifying the study as qualitative or indicating the approach (e.g., ethnography, grounded theory) or data collection methods (e.g., interview, focus group) is recommended | Above Line 1 |
|  | **Abstract** - Summary of key elements of the study using the abstract format of the intended publication; typically includes background, purpose, methods, results, and conclusions | 2 (Line 22 – 45) |
|  |  |  |
| **Introduction** | |  |
|  | **Problem formulation** - Description and significance of the problem/phenomenon studied; review of relevant theory and empirical work; problem statement | 3 – 5 (Line 48 – 109) |
|  | **Purpose or research questio**n - Purpose of the study and specific objectives or questions | 5 – 6 (Line 111 – 127) |
|  |  |  |
| **Methods** | |  |
|  | **Qualitative approach and research paradigm** - Qualitative approach (e.g., ethnography, grounded theory, case study, phenomenology, narrative research) and guiding theory if appropriate; identifying the research paradigm (e.g., postpositivist, constructivist/ interpretivist) is also recommended; rationale** | 6 (Line 131 – 135) |
|  | **Researcher characteristics and reflexivity** - Researchers’ characteristics that may influence the research, including personal attributes, qualifications/experience, relationship with participants, assumptions, and/or presuppositions; potential or actual interaction between researchers’ characteristics and the research questions, approach, methods, results, and/or transferability | 8 – 9 (Line 175– 177, 203 – 207) |
|  | **Context** - Setting/site and salient contextual factors; rationale** | 6 (Line 138 – 141) |
|  | **Sampling strategy** - How and why research participants, documents, or events were selected; criteria for deciding when no further sampling was necessary (e.g., sampling saturation); rationale** | 6 – 7 (Line 141 – 148) |
|  | **Ethical issues pertaining to human subjects** - Documentation of approval by an appropriate ethics review board and participant consent, or explanation for lack thereof; other confidentiality and data security issues | 6 (Line 144 – 145, 158 – 159) |
|  | **Data collection methods** - Types of data collected; details of data collection procedures including (as appropriate) start and stop dates of data collection and analysis, iterative process, triangulation of sources/methods, and modification of procedures in response to evolving study findings; rationale** | 7 – 8 (Line 162 – 164, 175 –181) |
|  | **Data collection instruments and technologies** - Description of instruments (e.g., interview guides, questionnaires) and devices (e.g., audio recorders) used for data collection; if/how the instrument(s) changed over the course of the study | 7 – 8 (Line 164 –171, 179) |
|  | **Units of study** - Number and relevant characteristics of participants, documents, or events included in the study; level of participation (could be reported in results) | 8 (Line 179 – 80), Table 1, Table 2 |
|  | **Data processing** - Methods for processing data prior to and during analysis, including transcription, data entry, data management and security, verification of data integrity, data coding, and anonymization/de-identification of excerpts | 7 (Line 156 – 157) |
|  | **Data analysis** - Process by which inferences, themes, etc., were identified and developed, including the researchers involved in data analysis; usually references a specific paradigm or approach; rationale** | 8 – 9 (Line 186 –199) |
|  | **Techniques to enhance trustworthiness** - Techniques to enhance trustworthiness and credibility of data analysis (e.g., member checking, audit trail, triangulation); rationale** | 9 (Line 201 – 207) |
|  |  |  |
| **Results/findings** | |  |
|  | **Synthesis and interpretation** - Main findings (e.g., interpretations, inferences, and themes); might include development of a theory or model, or integration with prior research or theory | 10 – 19 (Line 230 – 437) |
|  | **Links to empirical data** - Evidence (e.g., quotes, field notes, text excerpts, photographs) to substantiate analytic findings | 10 – 19 (Line 230 – 437) |
|  |  |  |
| **Discussion** | |  |
|  | **Integration with prior work, implications, transferability, and contribution(s) to the field -** Short summary of main findings; explanation of how findings and conclusions connect to, support, elaborate on, or challenge conclusions of earlier scholarship; discussion of scope of application/generalizability; identification of unique contribution(s) to scholarship in a discipline or field | 19 – 27 (Line 440 – 658) |
|  | **Limitations** - Trustworthiness and limitations of findings | 28 (Line 660 – 658) |
|  |  |  |
| **Other** | |  |
|  | **Conflicts of interest** - Potential sources of influence or perceived influence on study conduct and conclusions; how these were managed | 29 (Line 702) |
|  | **Funding** - Sources of funding and other support; role of funders in data collection, interpretation, and reporting | 29 (line 692 – 693) |
|  |  |  |
|  | *The authors created the SRQR by searching the literature to identify guidelines, reporting standards, and critical appraisal criteria for qualitative research; reviewing the reference lists of retrieved sources; and contacting experts to gain feedback. The SRQR aims to improve the transparency of all aspects of qualitative research by providing clear standards for reporting qualitative research. |  |
|  |  |  |
|  | **The rationale should briefly discuss the justification for choosing that theory, approach, method, or technique rather than other options available, the assumptions and limitations implicit in those choices, and how those choices influence study conclusions and transferability. As appropriate, the rationale for several items might be discussed together. |  |
|  |  |  |
|  | **Reference:** |  |
|  | O'Brien BC, Harris IB, Beckman TJ, Reed DA, Cook DA. **Standards for reporting qualitative research: a synthesis of recommendations.** *Academic Medicine*, Vol. 89, No. 9 / Sept 2014  DOI: 10.1097/ACM.0000000000000388 |  |
|  |  |  |
|  |  |  |

## Supplementary 3. Mapping of facilitators and barriers to TDF domains

| **TDF domains** | **Facilitators** | **Barriers** |
| --- | --- | --- |
| Skills | - | Lacked basic digital skills |
| Knowledge | - | Limited English literacy proficiency  Low education level |
| Memory, attention and decision processes | - | Anxiety-induced haste  Poor memory |
| Behavioural regulation | Integrated learned activities into existing exercise routines  Initiated meal planning and adopted a healthier dietary pattern | Inconsistent engagement with home-based exercises due to uncertainty about correct performance and reliance on external guidance |
| Emotion | - | Fear of fraudulent schemes  Fear of making mistakes  Stress and discouragement |
| Reinforcement | Awarded health points redeemable for transportation costs  Programmes and workshops were complimentary | - |
| Optimism | - | Pessimism  Self-ageist attitudes |
| Beliefs about capabilities | - | Low self-efficacy  Underlying health conditions (such as visual impairment and post-surgery) |
| Beliefs about consequences | Avoid health deterioration due to physical inactivity  Avoided long bank queues via using online banking  Gained benefits from online transactions  Reduced feelings of exclusion through digital competency  Increased awareness of fraud prevention | Pain exacerbation due to physical activity |
| Intentions | Intended to enhance knowledge  Intended to reinforce knowledge  Intended to keep up with technological advancements | - |
| Goals | Wanted to acquire knowledge  Wanted to learn new skills  Wanted to improve physical fitness (strength, balance and coordination) | - |
| Environmental context and resources | Accessible timing  Location convenience | Caregiving responsibility  Work schedule conflicts |
| Social influences | Encouragement from active ageing centres’ staff  Encouragement from friends | Discouragement from family members  Family concerns about fall risks  Tended to rely on family members to perform the task |
